# Genetic Diagnosis of Two Dopa-Responsive Dystonia Families by Exome Sequencing

**DOI:** 10.1371/journal.pone.0106388

**Published:** 2014-09-02

**Authors:** Zhan-fang Sun, Yu-han Zhang, Ji-feng Guo, Qi-ying Sun, Jun-pu Mei, Han-lin Zhou, Li-ping Guan, Jin-yong Tian, Zheng-mao Hu, Jia-da Li, Kun Xia, Xin-xiang Yan, Bei-sha Tang

**Affiliations:** 1 Department of Neurology, Xiangya Hospital, Central South University, Changsha, China; 2 Key Laboratory of Hunan Province in Neurodegenerative Disorders, Central South University, Changsha, China; 3 State Key Laboratory of Medical Genetics, Central South University, Changsha, China; 4 BGI-Shenzhen, Shenzhen, China; University of Southern California, United States of America

## Abstract

Dopa-responsive dystonia, a rare disorder typically presenting in early childhood with lower limb dystonia and gait abnormality, responds well to levodopa. However, it is often misdiagnosed with the wide spectrum of phenotypes. By exome sequencing, we make a rapid genetic diagnosis for two atypical dopa-responsive dystonia pedigrees. One pedigree, presented with prominent parkinsonism, was misdiagnosed as Parkinson's disease until a known mutation in *GCH1* (GTP cyclohydrolase 1) gene (NM_000161.2: c.631_632delAT, p.Met211ValfsX38) was found. The other pedigree was detected with a new compound heterozygous mutation in *TH* (tyrosine hydroxylase) gene [(NM_000360.3: c.911C>T, p.Ala304Val) and (NM_000360.3: c.1358G>A, p.Arg453His)], whose proband, a pregnant woman, required a rapid and less-biased genetic diagnosis. In conclusion, we demonstrated that exome sequencing could provide a precise and rapid genetic testing in the diagnosis of Mendelian diseases, especially for diseases with wide phenotypes.

## Introduction

Dopa-responsive dystonia (DRD) is a childhood-onset or adolescent-onset form of dystonia with excellent response to low dose levodopa [1]–[3]. To date, DRD has been reported to associate with mutations in genes encoding guanosine 5′-triphosphate (GTP) cyclohydrolase (GCH1), tyrosine hydroxylase (TH), and sepiapterin reductase (SPR) [4]–[6]. GCH1 and SPR are crucial enzymes for the biosynthesis of tetrahydrobiopterin (BH4), which serves as an essential cofactor for tyrosine and tryptophan hydroxylases in the initial biosynthesis of the neurotransmitter dopamine. TH is responsible for conversion tyrosine to L-dopa, a precursor of dopamine. Most cases present with the autosomal dominant form (*GCH1*-associated DRD), whereas the two recessively-inherited forms (*TH*-associated DRD and *SPR*-associated DRD) are rarer. Despite the availability of genetic testing, there still remains a marked delay in establishing the diagnosis with a delay of 15.2±13.7 years [2]. Differential diagnosis for some DRD patients is difficult with early-onset Parkinson's disease, cerebral palsy, spastic paraplegia, and early-onset primary dystonia, et al [7], [8]. Besides the wide spectrum of phenotypes, unknown genetic causes were found in five percent of DRD cases [9]. Despite the method of direct sequencing of coding regions is reasonable, PCR amplification of each candidate gene for phenotypes is cumbersome and incomprehensive. Exome sequencing, a combination of exon-capture and next generation sequencing technology, regarded as a rapid, cost-effective and comprehensive tool, has recently been successfully applied to genetic diagnosis of diseases [10], [11].

In this study, we made a rapid genetic diagnosis for two *GCH1*-associated DRD patients with prominent parkinsonism in one family, and two rarer *TH*-associated DRD siblings with long delay over 10 years from another unrelated family by exome sequencing. A previously reported heterozygous deletion mutation in the *GCH1* (c.631_632delAT, p.Met211ValfsX38), and two novel heterozygous missense mutations in the *TH* (c.911C>T, p.Ala304Val and c.1358G>A, p.Arg453His) were identified.

## Patients and Methods

### Patients

**Figure 1:**
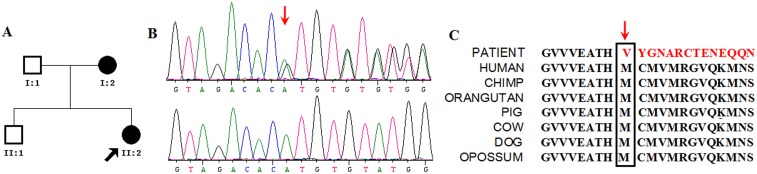
Identification of the (p.Met211ValfsX38) mutation in *GCH1* gene for family 1. (A) Pedigree of family 1 is indicated. Black and open symbols denote affected and unaffected individuals, respectively. The proband is indicated by an arrow. (B) Verification by Sanger sequencing of the frameshift mutation in *GCH1* gene (C) The mutation occurred at an evolutionarily conserved amino acid.

**Figure 2:**
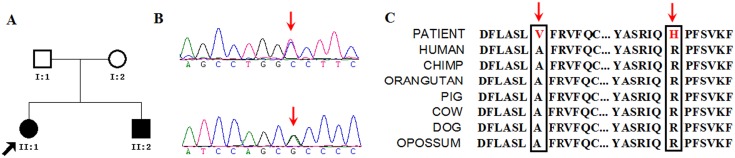
Identification of the (p.Ala304Val and p.Arg453His) mutations in *TH* gene for family 2. (A) Pedigree of family 2 is indicated. Black and open symbols denote affected and unaffected individuals, respectively. The proband is indicated by an arrow. (B) Verification by Sanger sequencing of the missense mutations in *TH* gene (C) The mutations occurred at two evolutionarily conserved amino acids.

Four DRD patients of Chinese Han ethnicity from two unrelated families were included in this study (Fig. 1A and Fig. 2A). All patients were subjected to a thorough neurological examination by two experienced neurologists in Xiangya Hospital affiliated to Central South University. Magnetic resonance imaging (MRI) of the brain showed no abnormalities for all patients. This study was approved by the ethics committee of Xiangya Hospital affiliated to Central South University. Written informed consent was obtained from each participant.

### Exome sequencing

The genomic DNA of four affected members in Family 1 and Family 2 were hybridized with Agilent SureSelect Human All Exon V1 and NimbleGen 2.1M-probe sequence capture array to enrich the exonic DNA, respectively. Exon-enriched DNA was sequenced by the Illumina (San Diego, CA) Hiseq 2000 platform following the manufacturer's instructions. Raw image files were processed by Illumina Pipeline (version 1.3.4) for base-calling with default parameters and the sequences of each individual were generated as 90 bp pair-end reads. The sequenced reads were aligned to the human reference genome (hg19 version) using bwa (0.5.9–r16). Bam files created by bwa were then processed GATK best practice pipeline using Genome Analysis ToolKit (GATK 2.8) to do the re-alignment and variation (SNV and Indel) detection, and Annovar was used to catalogue detected variations. Variations passed VQSR were extracted for the subsequent analysis. Like our previous criteria [12], [13], variations with homopolymer length >6 or common (>0.5%) in HapMap, 1000 Genome Project, dbSNP (v137), and our in-house variant database (unrelated healthy Chinese subjects, BGI-Shenzhen, China) were then discarded. Finally, non-synonymous/splicing/frameshift (NS/S/F) variations evaluated deleterious or possible deleterious by Sorting Intolerant from Tolerant (SIFT), Polymorphism Phenotyping (PolyPhen-2), or MutationTaster were considered as candidate causal variations. We have shared our deep-sequencing data in NCBI Short Read Archive and the accession number is SRP043259.

### Multiple ligation-dependent probe amplification (MLPA)

We performed a multiplex ligation-dependent probe amplification (MLPA) assay targeting *GCH1* using SALSA kit P099-C2 (MRC-Holland, Amsterdam) for the four cases from two families. This probemix contains probes for all 6 exons of the *GCH1* gene, 6 out of the 14 exons of the *TH* gene and an additional flanking probe and all 11 *SGCE* exons present in NM_003919.2. MLPA amplification products were separated on a capillary sequencer (ABI PRISM 3100 Genetic Analyzer). MLPA data were analyzed by Coffalyser (MRC-Holland, Amsterdam). Deletions were considered in exons when the relative peak area was reduced by approximately 50% of the normal control values.

### Plasmids construction, Eukaryotic expression, and Assay of TH activity

A wild-type human *TH* sequence fragment cDNA (NM_000360) was subcloned into the pYr-ads-1-flag-N vector at EcoRI/BamHI sites. Mutant TH^A304V^ and TH^R453H^ were generated with QuikChange site-directed mutagenesis protocol (YRGene, China). The primers used in this study are shown in Table S1 of File S1. All constructs were confirmed by sequencing. The human embryonic kidney 293 (HEK293) cell was transfected with wild-type and mutant vector DNA using lipofectamine (GIBCO-BRL). Cells were harvested after 40 h, lysed by sonication, centrifuged (15,000 g for 15 min at 4°C) and the supernatant was kept at −80°C until use. 100 µl of supernatant was reacted chemically with 1 ml of mixed liquor (0.3 mmol/L L-Tyrosine, 0.08 mmol/L Tetrahydro-L-Biopterin, and 0.1 mmol/L FeSO_4_) at 37°Cfor 10 min, terminated by 100 µl of perchloric acid [14], [15]. The product for L-dihydroxyphenylalanine (L-DOPA) was detected by high performance liquid chromatography (HPLC). Briefly, chromatographic separation was performed on a Waters Symmetry C18 column (150 mm×4.6 mm, 5 µm, Milford, MA, USA) in tandem with a Phenomenex C18 guard cartridge (4.0 mm×3.0 mm, Phenomenex, Torrance, CA). The eluent was delivered from an Agilent 1260 HPLC quaternary pump (Agilent Technologies, Santa Clara, CA) equipped with an online vacuum degasser, an autosampler, a thermostated column compartment, and a diode array detector. The mobile phase was a methanol/0.01 mol/L phosphate buffer (V/V = 4/96) mixture with pH 3.0. The flow rate was set at 0.6 ml/min while the temperature was controlled at 25°C. Spectra were acquired at 280 nm. The specific enzyme activity of TH [L-DOPA (nmol)/min/TH (ng)] was normalized with the amount of TH protein, which was quantified using ELISA (Uscn Life Science Inc) as described by the manufacturer. Sample analyses were performed in three independent experiments.

## Results

### Clinical presentation

In family 1, the index case (II-2), a 22-year-old female patient with bilateral hand tremor, postural instability and gait difficulties, was initially diagnosed as early-onset Parkinson's disease when she was 13. Since then, she has been taking levodopa at 250 mg/day, sustaining a good response. Her mother (I-2), a 53-year-old patient, with rest tremor and slight bradykinesia for right hand for 5 years, was initially diagnosed as adult-onset Parkinson's disease. She was treated with levodopa/pramipexole at 500 mg/0.5 mg daily and had a good response. To identify potential causative genetic variants in this pedigree, we performed exome sequencing on these two affected individuals (I-2 and II-2 in Fig. 1A).

In family 2, the index case was II-1, a 23-year-old female patient. At age 3, she suffered with gait disturbance characterized by left leg stiffness and a tendency to walk with an equinus gait. She received physiotherapy and multiple antispasticity medications with no benefit. The symptoms, including the postural tremor of the limbs, upper limb stiffness, and torticollis, appeared at age 22. A diagnosis of DRD was suspected. She was treated with levodopa at 500 mg/day for two days with good response. Since then, she has been taking levodopa at 250 mg/day, even during the period of pregnancy, sustaining an excellent response. Her brother (II-2), a 13-year-old patient, presented with gait disturbance and generalized rigidity since 3 years old, was treated with levodopa at 250 mg/day with an excellent response after the symptoms of his sister had a good response to levodopa. To make a rapid and comprehensive genetic diagnosis, the index case with 4 months of pregnancy, was admitted to us. Exome sequencing was performed on her and her affected brother (II-1 and II-2 in Fig. 2A).

### Exome Sequencing

Exome sequencing was performed for the four affected individuals. Sequencing Quality (≥20) was greater than 90%. Coverage of target region for each of four samples was over 97%, and about 90% of the exome was covered >10×. An average of 6.34 and 6.17 gigabases (Gb) of sequence were generated per affected individual for family 1 and family 2, respectively (Table S2 of File S1). The variant data were next filtered to allow removal of likely benign variants in HapMap, 1000 Genome Project, dbSNP (v137), and our in-house variant database (unrelated healthy Chinese subjects) with a minor allele frequency>0.5%. Less than 200 novel or rare variants were found per individual. Variants were further filtered to predict as damaging by SIFT, PolyPhen-2, or MutationTaster.

For family 1, considering the difference of phenotype between two affected individuals, we categorized the predicted damaging variants on the basis of the following criteria: 1. 68 variants shared by two individuals (Table S3 of File S1); 2. 35 variants exclusive to the patient I-2 (Table S4 of File S1); 3. 37 variants exclusive to the patient II-2 (Table S5 of File S1). For these 140 variants, priority was given to variants belonging to genes known to be associated with a special phenotype in OMIM. After that, we identified 30 damaging variants (in 28 genes) in either of two patients or both. Inspection of these 30 variants revealed a previously described pathogenic mutation (g.55310856delAT, c.631_632delAT, p.Met211ValfsX38) in *GCH1* associated with DRD, while the other 29 variants were not known to be associated with parkinsonism. The GCH1 has one domains: GTP cyclohydrolase I (GTP_cyclohydro I) which catalyses the biosynthesis of formic acid and dihydroneopterin triphosphate from GTP and locates at amino acids 71–249 (http://pfam.sanger.ac.uk), thus, the mutation locates in the domain of GTP_cyclohydro I of *GCH1*. The mutation was confirmed in the affected individuals (Fig. 1B) and not found in the unaffected family members by Sanger sequencing. Thus, the mutation in GCH1 completely cosegregated with the phenotype in this family. We found that the variant resulted in frameshift mutation and a truncated protein, occurred in a highly conserved position (Fig. 1C), and was predicted to be disease-causing. To explain intrafamilial phenotypic variability in Family 1, we further performed functional categories of genes in the different damaging variants between two patients with DAVID [16]. However, no functional categories are enriched after multiple comparison correction for two patients (Table S6 of File S1).

For family 2, in accordance with the pedigree, we assumed that both patients should show NS/S/F variants in the same gene. After filtering databases and further predicting damaging variants, 101 variants were shared by both affected siblings, including 3 homozygous variants, 17 compound heterozygous variants, and 81 heterozygous variants. Of these variants, 42 variants in 38 genes were associated with a special phenotype in OMIM (Table S7 of File S1). According to the pedigree of this family, recessive inheritance model was the highest priority. Thus, we focused on the 3 homozygous and 17 compound heterozygous variants (in 10 genes). After that, *TH* gene which has known to associate with DRD was identified. Since we could not absolutely exclude the possibility of dominant inheritance for this DRD pedigree, we also analyzed the 81 heterozygous variants except for the 17 compound heterozygous variants, and identified no other compelling candidate variants for disease-causing mutations for family 2. The TH has four domains: three Tyrosine hydroxylase N terminal (TOH_N) domains and one Biopterin-dependent aromatic amino acid hydroxylase (Biopterin_H) domain which is rate-limiting catalyst for many important metabolic pathways and locates at amino acids 195–526 (http://pfam.sanger.ac.uk). Two novel heterozygous missense mutations (g. 2187929C>T, c.911C>T, p.Ala304Val and g. 2185599G>A, c.1358G>A, p.Arg453His) in domain of Biopterin_H of *TH* were identified and not reported in HGMD Database (http://www.hgmd.cf.ac.uk). Both mutations were confirmed as compound heterozygous mutations in the affected siblings (Fig. 2B) and as heterozygous state in their parents by Sanger sequencing. We found that the both variants occurred in a highly conserved position (Fig. 2C) and were predicted to be disease-causing. Additionally, both mutations were not detected in 300 unaffected controls by Sanger sequencing.

**Figure 3:**
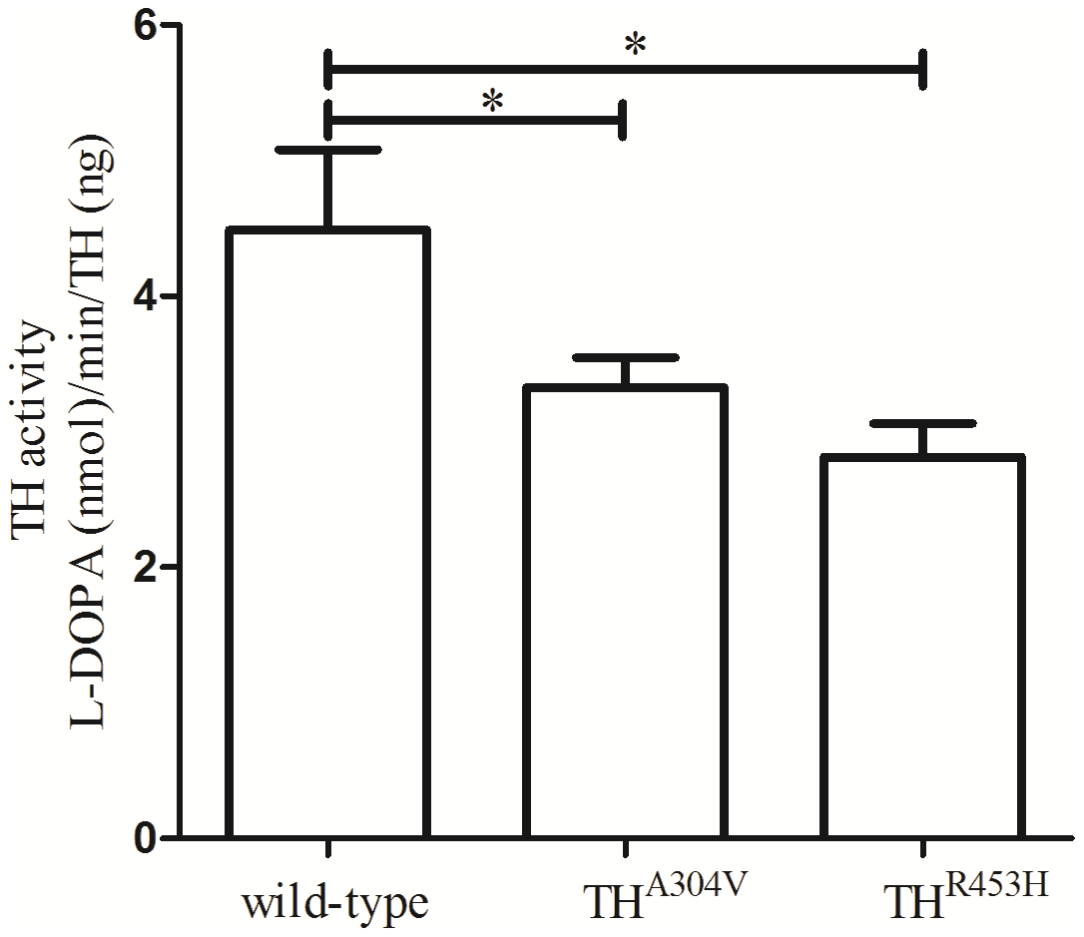
The specific TH activity of the cell lysates after transfection with wild-type TH, mutant TH^A304V^, and TH^R453H^. Compared with the wild-type, the specific TH activity with the mutant TH^A304V^ and TH^R453H^ were both significantly decreased (*P*<0.05). The average levels of TH activity were plotted, and error bars correspond to the standard deviation (SD). The *t* tests were used for statistics.

After a genetic diagnosis of DRD for the two families, we further performed a MLPA assay to detect deletions/duplications of one or more sequences in the *GCH1* and *TH* genes. And no exon deletion/duplication of the *GCH1* and *TH* gene was detected for four cases in the two families (MLPA profile in Fig. S1). To further demonstrate the possibility of damaging of the new compound heterozygous mutations in *TH* gene for family 2, we transfected HEK293 cells with the wild-type and mutant cDNAs. Then, transiently expressed TH was analyzed for activity. The specific TH activity of the cell lysates after transfection with the mutant TH^A304V^ and TH^R453H^ were decreased to 74.1% and 62.7% of the wild-type (*P* = 0.03 and *P* = 0.01, respectively) (Fig. 3).

## Discussion

This work demonstrates the application of whole-exome sequencing to a precise genetic testing in two DRD pedigrees. The two patients were initially diagnosed as PD in the family 1, and the diagnosis of DRD or other diseases causing juvenile parkinsonism and dystonia for the index were considered in the referral differential diagnosis. The detection of a known mutation c.631_632delAT in *GCH1* supported the unexpected diagnosis of DRD for the two patients. The frameshift mutation was firstly reported in a sporadic DRD case without description of parkinsonism [17]. Afterwards, three female patients with this frameshift mutation manifested generalized or hemi-dystonia were reported [9]. In contrast, two patients in family 1 showed prominent parkinsonism though there was slight dystonia of lower limbs for the index case. When the clinical diagnosis was re-evaluated, additional evidence in favor of DRD for the index case is that the patient did not develop dyskinesia even after 9 years of levodopa treatment, while levodopa-induced dyskinesia usually occur early in the course of levodopa treatment in the early-onset PD patients [18]. Nevertheless, it was very difficult for the differential diagnosis between DRD, early-onset PD and other diseases with juvenile parkinsonism and dystonia without genetic testing for this index case. The early-onset PD patients with homozygous or compound heterozygous mutations in *parkin* gene, for instance, also manifested slowly progressive, levodopa-responsive parkinsonism without uncommon dystonia of lower limbs, and the parents with heterozygous *parkin* mutations, sometimes, suffered from adult-onset Parkinson disease [19]. Indeed, some DRD patients presented with a combination of dystonic and parkinsonian signs have been described [20], [21]. However, the diagnosis of DRD in patients only with parkinsonism like the mother of index case (I-2) was rarely reported. A 33-year-old Chinese DRD patient with pure parkinsonism were described, who had a heterozygous mutation in *GCH1* gene [22]. Another patient was a 65-year-old woman harbouring a heterozygous *GCH1* deletion, who showed mild parkinsonian symptoms without signs of dystonia, and her daughter carrying the same *GCH1* heterozygous mutation was suffered from DRD [23]. Additionly, there were a few reports about adult-onset DRD patients presented with pure parkinsonism in autosomal dominant DRD pedigrees though the genetic testing of *GCH1* gene was not available at that time [24], [25]. These observations suggested that there might be a specific subgroup of DRD with pure parkinsonism and *GCH1* mutation. We recommend that genetic counselling and testing for *GCH1* should not be neglected in patients with pure parkinsonism.

We described two patients in a DRD kindred harbouring the same mutation with distinct clinical presentations. We performed literature retrieval and analysis of clinical phenotype of all damaging variants for two patients. No other damaging variants associated with parkinsonism or dystonia were found. After functional analysis of different damaging variants between two patients, however, no functional categories were enriched. Nonetheless, a possible role of some modulatory genes for the individual phenotype in the family 1 was not completely excluded. Previous attempts to explain intrafamilial phenotypic variability in carriers with the same mutation include the observation that carriers may have different mutant/wild type GCH1 messenger RNA ratios [26]. However, it is not certain whether the ratio in lymphocytes can actually reflect that in the brain. Other possible explanations included post-translational modifications, environmental factors, et al [26], [27]. The mechanisms underlying the phenomenon of variable penetrance are likely to be complicated, and more pedigrees with same mutation but phenotypic variability should be further studied.

For family 2, we could not absolutely exclude the possibility of dominant inheritance for this DRD pedigree, considering the low penetrance of the DRD-associated genes. The analysis of recessive inheritance model, however, should be the highest priority according to the pedigree of this family. Whole-exome sequencing of both siblings implicated 10 genes with 3 homozygous and 17 compound heterozygous variants, which are potentially causative for the siblings. Only one of these genes, *TH*, was a high-priority candidate by functional criteria. This gene has been implicated previously in DRD. The missense mutations [(p.Ala304Val) and (p.Arg453His)] affect two amino acid residues in the Biopterin_H domain, which are highly conserved positions, predicted to be disease-causing. Indeed, we also tried to analysis results of candidate genes obtained from exome sequencing under a dominant inheritance model for family 2 and no other genes associated with dystonia or parkinsonism were found. To further demonstrate the possibility of damaging of the new compound heterozygous mutations, we studied TH activity in vitro experiment. The specific TH activity of the cell lysates after transfection with the mutant TH^A304V^ and TH^R453H^ were both significantly decreased compared with the wild-type, which also suggested a disease-causing mutations.

One could argue it was, more or less, costly way to identify the mutation of known DRD-causing *TH* gene by exome sequencing. However, exome sequencing in this work provided rapid genetic testing and accounted for the clinical diagnosis for two siblings after long-term misdiagnoses in the family 2. And we could not absolutely exclude the possibilities of *SPR* gene or *GCH1* gene mutations before this study according to their clinical symptoms and a family history. Further, a DRD phenotype caused by *parkin* gene or other genes involved in pterin metabolism were also reported [9], [28]. Although by candidate gene sequencing *TH* mutations would have been identified successfully in this case, genetic testing for potential gene does not eliminate other genes and becomes time-consuming for the index case, and not directed by clinically observed phenotypic information. In fact, most *TH*-DRD patients developed severe motor retardation and hypokinesia rather than typical dystonia [26]. Exome sequencing has the advantage of being less biased and saving time compared with candidate-gene sequencing, with the further developments in the production and interpretation of data, which is anticipated to be used increasingly in molecular diagnosis [29], [30].

In conclusion, we have demonstrated that exome sequencing of a small number of affected family members is a powerful, efficient, and cost-effective strategy for markedly reducing the pool of candidate genes for Mendelian diseases, and may even identify the responsible gene specifically. With falling of costs, this approach will likely become part of the routine clinical evaluation of patients with suspected genetic diseases in whom the diagnosis is not very certain.

## Supporting Information

Figure S1 **MLPA profile.** Multiplex ligation-dependent probe amplification (MLPA) profile of four patients in the two families showed no exon deletion/duplication of the *GCH1*, *TH*, and *SGCE* gene.
(TIF)

File S1 **Supporting tables.** Table S1, The primers used for cDNA amplification of *TH*. Table S2, Exome Sequencing Statistics for four patients. Table S3, Damaging variants predicted in silico detected by exome sequencing and shared between individual I-2 and II-2 in family 1. Table S4, Damaging variants predicted in silico detected by exome sequencing and were exclusively for the patient I-2. Table S5, Damaging variants predicted in silico detected by exome sequencing and were exclusively for the patient II-2. Table S6, Functional enrichment for patients (I-2 and II-2) in family 1. No functional categories are enriched after multiple comparison correction. Table S7, Analytic results of candidate variants obtained from exome sequencing for family 2.
(DOC)
