# Supplementary figures and images for: Genetic Diagnosis of Two Dopa-Responsive Dystonia Families by Exome Sequencing
Source: PLoS One. 2014 Sep 2;9(9):e106388. doi: 10.1371/journal.pone.0106388 (PMC4152247; doi:10.1371/journal.pone.0106388)

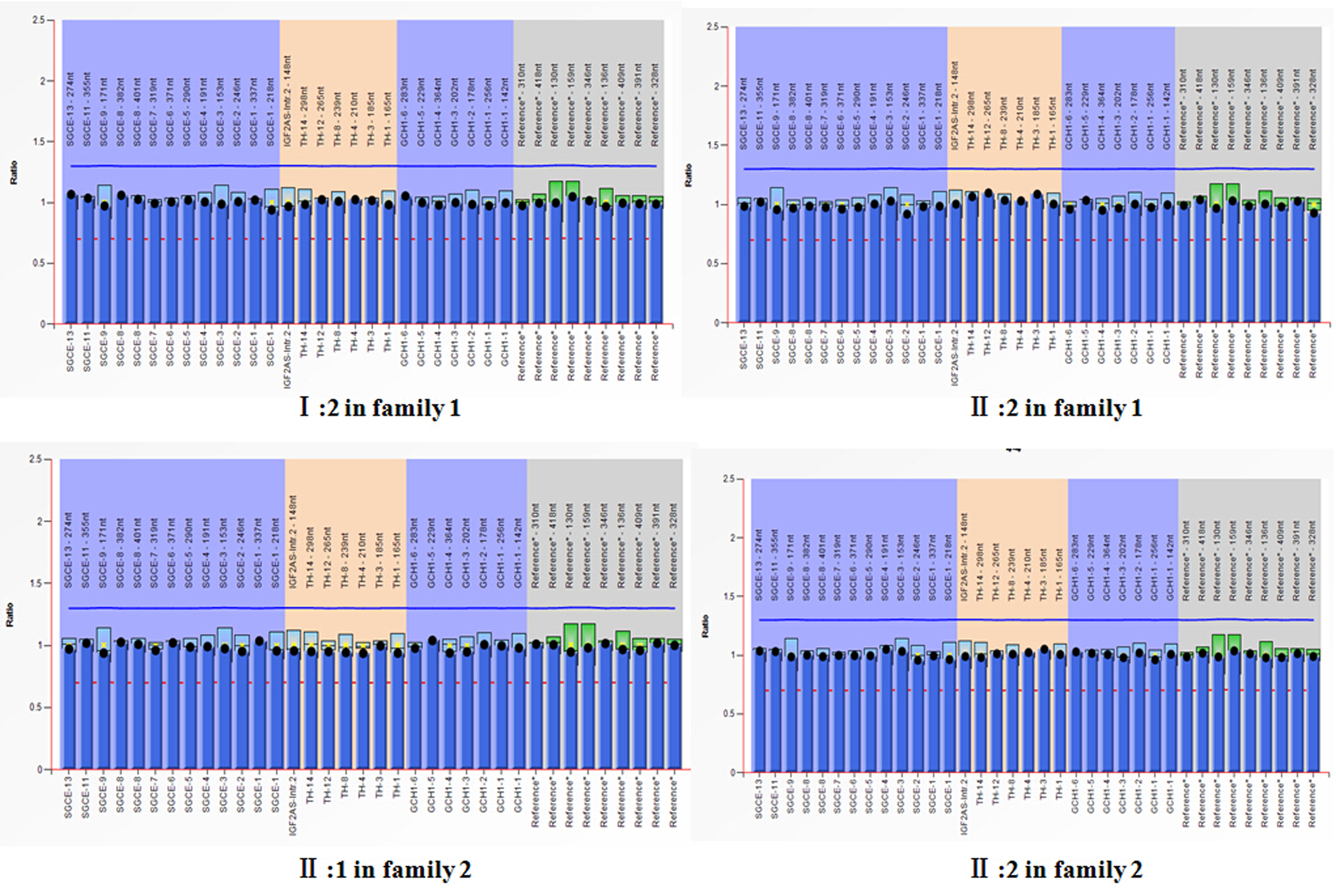

Supplement: Figure S1 — MLPA profile. Multiplex ligation-dependent probe amplification (MLPA) profile of four patients in the two families showed no exon deletion/duplication of the GCH1, TH, and SGCE gene. (TIF) [file pone.0106388.s001.tif]
