# Supplementary material for: Genetic Diagnosis of Two Dopa-Responsive Dystonia Families by Exome Sequencing
Source: PLoS One. 2014 Sep 2;9(9):e106388. doi: 10.1371/journal.pone.0106388 (PMC4152247; doi:10.1371/journal.pone.0106388)
Supplement: File S1 — Supporting tables. Table S1, The primers used for cDNA amplification of TH. Table S2, Exome Sequencing Statistics for four patients. Table S3, Damaging variants predicted in silico detected by exome sequencing and shared between individual I-2 and II-2 in family 1. Table S4, Damaging variants predicted in silico detected by exome sequencing and were exclusively for the patient I-2. Table S5, Damaging variants predicted in silico detected by exome sequencing and were exclusively for the patient II-2. Table S6, Functional enrichment for patients (I-2 and II-2) in family 1. No functional categories are enriched after multiple comparison correction. Table S7, Analytic results of candidate variants obtained from exome sequencing for family 2. (DOC) [file pone.0106388.s002.doc]

**Supplementary Information:**

**Table S1.** **The** **primers used for cDNA amplification of *TH***

| wide-type or mutant TH | primers |
| --- | --- |
| wide-type | F: CGGGATCCGCCACCATGCCCACCCCCGACGCCACCA |
| wide-type | R: GGAATTCGCCAATGGCACTCAGCGCAT |
| A304V | F: GACTTCCTGGCCAGCCTGGTCTTCCGCGTGTTCCAGTGCA |
| A304V | R: TGCACTGGAACACGCGGAAGACCAGGCTGGCCAGGAAGTC |
| R453H | F: TATGCCTCACGCATCCAGCACCCCTTCTCCGTGAAGTTC |
| R453H | R: GAACTTCACGGAGAAGGGGTGCTGGATGCGTGAGGCATA |

**Table S2. Exome-Sequencing Statistics for four patients**

| Sample | I -2 in family 1 | Ⅱ-2 in family 1 | Ⅱ-1 in family 2 | Ⅱ-2 in family 2 |
| --- | --- | --- | --- | --- |
| Clean Yield(Gb) | 6.17 | 6.50 | 6.17 | 6.17 |
| Sequencing Quality >= 20 | 95.18% | 96.06% | 93.83% | 94.37% |
| Average sequencing depth on target | 78.43 | 89.20 | 78.62 | 75.26 |
| Coverage of target region | 98.40% | 98.80% | 97.50% | 97.70% |
| Fraction of target covered with at least 20x | 78.30% | 83.40% | 78.80% | 78.80% |
| Fraction of target covered with at least 10x | 87.90% | 91.50% | 87.60% | 87.90% |
| Fraction of target covered with at least 4x | 94.70% | 96.30% | 93.70% | 94.10% |
| Mapping rate | 99.35% | 99.40% | 99.07% | 98.92% |

**Table S3.**

**Damaging variants predicted in silico detected by exome sequencing and shared between individual I -2 and Ⅱ-2 in family 1**

| **Chromosome** | **Position** | **Gene Name** | **Substitution** | **Gene-Phenotype by OMIM** | **Score from SIFT, Polyphen-2, and MutationTaster** |
| --- | --- | --- | --- | --- | --- |
| chr14 | 55310856 | ***GCH1*** | delAT | Dystonia, DOPA-responsive, with or without hyperphenylalaninemia; Hyperphenylalaninemia, BH4-deficient, B | NA, NA, 1 (D) |
| chr1 | 143767547 | *PPIAL4G* | G>A | None | 0.03, 0.999, 0.079 (P) |
| chr1 | 145769496 | *GPR89A* | A>C | None | 0.06, 0.999, 1 (D) |
| chr1 | 154541974 | *CHRNB2* | A>G | Epilepsy, nocturnal frontal lobe, 3 | 0.03, 0.557, 1 (D) |
| chr1 | 169811573 | *C1orf112* | G>C | None | 0.05, 0.95, 1 (D) |
| chr1 | 222837358 | *MIA3* | G>A | None | 0.34, 1, 1 (D) |
| chr2 | 74184283 | *DGUOK* | G>T | Mitochondrial DNA depletion syndrome 3 | 0, 1, 1 (D) |
| chr2 | 109276099 | *LIMS1* | G>A | None | 0.05, 0.999, 1 (D) |
| chr2 | 120223434 | *SCTR* | A>G | None | 0.04, 0.766, 1 (D) |
| chr2 | 131117010 | *PTPN18* | C>T | None | 0, 1, 0 (D) |
| chr2 | 179633421 | *TTN* | T>G | Cardiomyopathy, dilated, 1G; Cardiomyopathy, familial hypertrophic, 9 | 0, 0.993, 0.002 (P) |
| chr2 | 220100503 | *ANKZF1* | G>A | None | 0.06, 0.988, 0.328 (P) |
| chr3 | 44795728 | *KIAA1143* | C>G | None | 0.22, 0.98, 0.569 (D) |
| chr3 | 44816862 | *KIF15* | G>A | None | 0.65, 0.999, 1 (D) |
| chr3 | 49167685 | *LAMB2* | G>A | Nephrotic syndrome, type 5, with or without ocular abnormalities; Pierson syndrome | 0.05, 1, 1 (D) |
| chr3 | 58116605 | *FLNB* | C>T | Atelosteogenesis, type I; Atelosteogenesis, type III; Boomerang dysplasia; Larsen syndrome; Spondylocarpotarsal synostosis syndrome | 0.04, 1, 1 (D) |
| chr3 | 101066862 | *SENP7* | G>T | None | 0.01, 1, 0.863 (D) |
| chr3 | 101572638 | *NFKBIZ* | A>G | None | 0.19, 0.998, 1 (D) |
| chr3 | 184428924 | *MAGEF1* | A>C | None | 0, 0.996, 0.897 (D) |
| chr4 | 2242253 | *HAUS3* | C>T | None | 1, 0.892, 0 (P) |
| chr4 | 8407766 | *ACOX3* | C>G | None | 0, 1, 1 (D) |
| chr4 | 141789449 | *RNF150* | G>C | None | 0.01, 0.999, 0.999 (D) |
| chr5 | 83476265 | *EDIL3* | T>C | None | 0.24, 0.743, 1 (D) |
| chr5 | 140186980 | *PCDHA4* | G>A | None | 0.03, 0.467, 0 (P) |
| chr5 | 140263497 | *PCDHA13* | C>G | None | 0.01, 0.999, 0.127 (P) |
| chr6 | 136582417 | *BCLAF1* | G>A | None | 0.04, 1, 1 (D) |
| chr6 | 151789965 | *C6orf211* | C>T | None | 0.22, 1, 1 (D) |
| chr7 | 76254940 | *POMZP3* | A>C | None | 0, 0.88, 0.001 (D) |
| chr7 | 151970931 | *KMT2C* | G>A | None | 0.14, 1, 1 (D) |
| chr8 | 52733242 | *PCMTD1* | C>T | None | 0.07, 1, 1 (D) |
| chr8 | 65517387 | *CYP7B1* | A>T | Bile acid synthesis defect, congenital, 3; Spastic paraplegia 5A, autosomal recessive | 0, 1, 0.313 (P) |
| chr8 | 101718965 | *PABPC1* | G>A | None | 0.03, 0.97, 1 (D) |
| chr8 | 103664436 | *KLF10* | T>G | None | 001, 0.641, 0 (P) |
| chr9 | 4666216 | *SPATA6L* | C>T | None | 0.14, 1, 1 (D) |
| chr9 | 107599738 | *ABCA1* | T>G | HDL deficiency, type 2; Tangier disease | 0.05, 0.988, 1 (D) |
| chr9 | 131243911 | *ODF2* | A>G | None | 0.02, 0.962, 1 (D) |
| chr10 | 117075092 | *ATRNL1* | C>G | None | 0, 1, 1 (D) |
| chr11 | 47188409 | *ARFGAP2* | G>A | None | 0.01, 0.999, 0.865 (D) |
| chr11 | 120097553 | *OAF* | A>G | None | 0, 1, 1 (D) |
| chr12 | 11183793 | *TAS2R31* | G>C | None | 0.05, 0.75, 0 (P) |
| chr12 | 20786694 | *PDE3A* | C>T | None | 0.12, 0.996, 0 (P) |
| chr12 | 22676453 | *C2CD5* | A>C | None | 0, 1, 1 (D) |
| chr12 | 48143751 | *RAPGEF3* | A>C | None | 0, 1, 1 (D) |
| chr13 | 25021263 | *PARP4* | T>C | None | 0, 0.997, 0.948 (D) |
| chr14 | 23444185 | *AJUBA* | G>T | None | 0.04, 0.298, 1 (D) |
| chr14 | 31604298 | *HECTD1* | G>A | None | 0, 1, 1 (D) |
| chr15 | 28566548 | *HERC2* | T>C | Mental retardation, autosomal recessive 38 | 0.15, 0.963, 0.997 (D) |
| chr16 | 68349941 | *PRMT7* | A>G | None | 0.06, 0.999, 0.532 (D) |
| chr17 | 1387495 | *MYO1C* | C>T | None | 0, 1, 1 (D) |
| chr17 | 5037259 | *USP6* | C>G | None | 0.98, 0.986, 1 (D) |
| chr17 | 6021383 | *WSCD1* | A>G | None | 0.19, 0.998, 1 (D) |
| chr17 | 16068340 | *NCOR1* | C>T | None | 0.03, 1, 1 (D) |
| chr17 | 16068377 | *NCOR1* | C>G | None | 0, 1, 1 (D) |
| chr17 | 45214690 | *CDC27* | G>A | None | 0, 1, 1 (D) |
| chr17 | 45234707 | *CDC27* | T>A | None | 0.07, 0.624, 1 (D) |
| chr17 | 46239878 | *SKAP1* | C>T | None | 0.01, 0.999, 1 (D) |
| chr17 | 65026687 | *CACNG4* | C>G | None | 0.03, 1, 1 (D) |
| chr18 | 42281774 | *SETBP1* | G>A | Schinzel-Giedion midface retraction syndrome | 0.15, 0.971, 0.983 (D) |
| chr19 | 6697740 | *C3* | G>T | C3 deficiency; Hemolytic uremic syndrome, atypical, susceptibility to, 5 | 0, 1, 1 (D) |
| chr19 | 36370001 | *APLP1* | G>A | None | 0.1, 0.988, 1 (D) |
| chr20 | 60848375 | *OSBPL2* | C>G | None | 0.03, 1, 1 (D) |
| chr21 | 40777903 | *LCA5L* | A>G | None | 0.01, 0.999, 0.178 (P) |
| chr22 | 24579157 | *SUSD2* | G>T | None | 0.31, 1, 1 (D) |
| chr22 | 26830276 | *ASPHD2* | A>G | None | 0.05, 0.97, 1 (D) |
| chr1 | 248458133 | *OR2T12* | delAA | None | NA, NA, 1 (D) |
| chr8 | 142161936 | *DENND3* | insGTTA | None | NA, NA, 1 (D) |
| chr12 | 52967189 | *KRT74* | delG | Hypotrichosis simplex of the scalp 2; Woolly hair, autosomal dominant | NA, NA, 1 (D) |
| chr15 | 42436671 | *PLA2G4F* | delC | None | NA, NA, 1 (D) |

Sift score≤0.05 indicates deleterious; Polyphen-2 score≥0.453 indicates damaging; NA, not available; D, disease-causing predicted by Mutationtaster; P, polymorphism predicted by Mutationtaster.

**Table S4.**

**Damaging variants predicted in silico detected by exome sequencing and were exclusively for the patientⅠ-2**

| **Chromosome** | **Position** | **Gene Name** | **Substitution** | **Gene-Phenotype by OMIM** | **Score from SIFT, Polyphen-2, and MutationTaster** |
| --- | --- | --- | --- | --- | --- |
| chr1 | 41304121 | *KCNQ4* | G>A | Deafness, autosomal dominant 2A | 0.06, 0.984, 0.991 (D) |
| chr1 | 43916931 | *HYI* | C>A | None | 0.01, 0.993, 1 (D) |
| chr1 | 62271183 | *INADL* | A>G | None | 0.26, 0.995, 1 (D) |
| chr1 | 152276459 | *FLG* | C>T | Ichthyosis vulgaris | 0.44, 0.996, 0 (P) |
| chr1 | 169484750 | *F5* | T>G | Factor V deficiency; Thrombophilia due to activated protein C resistance | 0.1, 0.739, 0 (P) |
| chr1 | 169823592 | *SCYL3* | A>T | None | 0, 1, 1 (D) |
| chr2 | 9618476 | *IAH1* | G>A | None | 0, 1, 1 (D) |
| chr2 | 114210728 | *CBWD2* | C>T | None | 0.01, 1, 1 (D) |
| chr2 | 198261032 | *SF3B1* | G>T | Myelodysplastic syndrome, somatic | 0, 0.997, 1 (D) |
| chr3 | 16237352 | *GALNT15* | C>T | None | 0, 1, 1 (D) |
| chr3 | 97868441 | *OR5H14* | C>G | None | 0, 0.761, 0 (P) |
| chr3 | 98002036 | *OR5H2* | G>T | None | 0, 0.999, 1 (D) |
| chr5 | 34830853 | *RAI14* | C>T | None | 0.04, NA, 1 (D) |
| chr8 | 133141573 | *KCNQ3* | G>A | Seizures, benign neonatal, type 2 | 0, 0.987, 1 (D) |
| chr9 | 97084600 | *NUTM2F* | C>T | None | 0, 1, 0 (P) |
| chr9 | 101304301 | *GABBR2* | C>A | Nicotine dependence, protection against | 0.2, 0.999, 1 (D) |
| chr11 | 17809916 | *SERGEF* | C>T | None | 0, 1, 1 (D) |
| chr11 | 21250976 | *NELL1* | G>T | None | 0.06, 0.989, 0.999 (D) |
| chr11 | 64374905 | *NRXN2* | G>T | None | 0.01, 1, 1 (D) |
| chr12 | 32949200 | *PKP2* | T>C | Arrhythmogenic right ventricular dysplasia 9 | 0.04, 0.01, 0.231 (P) |
| chr12 | 53897517 | *TARBP2* | C>T | None | 0.04, 0.995, 0.999 (D) |
| chr15 | 28483903 | *HERC2* | C>A | Mental retardation, autosomal recessive 38 | 0.07, 0.964, 1 (D) |
| chr16 | 816142 | *MSLN* | G>A | None | 0.06, 1, 0.011 (P) |
| chr16 | 14988897 | *NOMO1* | G>C | None | 0.21, 0.999, 1 (D) |
| chr16 | 15703403 | *KIAA0430* | C>T | None | 0.09, 0.999, 1 (D) |
| chr16 | 87744968 | *KLHDC4* | T>G | None | 0.14, 0.925, 0 (P) |
| chr16 | 89661944 | *CPNE7* | G>A | None | 0.12, 1, 1 (D) |
| chr17 | 37881634 | *ERBB2* | C>T | Adenocarcinoma of lung, somatic; Gastric cancer, somatic; Glioblastoma, somatic; Ovarian cancer, somatic | 0, NA, 1 (D) |
| chr17 | 39197186 | *KRTAP1-1* | C>T | None | 0.02, 1, 0.979 (D) |
| chr20 | 56140567 | *PCK1* | C>T | Phosphoenolpyruvate carboxykinase-1, cytosolic, deficiency | 0, 0.999, 1 (D) |
| chr22 | 45937184 | *FBLN1* | C>T | Synpolydactyly, 3/3'4, associated with metacarpal and metatarsal synostoses | 0.09, 1, 0.963 (D) |
| chr1 | 152284041 | *FLG* | delT | Ichthyosis vulgaris | NA, NA, 1 (D) |
| chr17 | 67184001 | *ABCA10* | delTT | None | NA, NA, 1 (D) |
| chr19 | 44740871 | *ZNF227* | delAG | None | NA, NA, 1 (D) |
| chrX | 22245728 | *PHEX* | insGTGA | Hypophosphatemic rickets, X-linked dominant | NA, NA, 1 (D) |

Sift score≤0.05 indicates deleterious; Polyphen-2 score≥0.453 indicates damaging; NA, not available; D, disease-causing predicted by Mutationtaster; P, polymorphism predicted by Mutationtaster.

**Table S5.**

**Damaging variants predicted in silico detected by exome sequencing and were exclusively for the patient Ⅱ-2**

| **Chromosome** | **Position** | **Gene Name** | **Substitution** | **Gene-Phenotype by OMIM** | **Score from SIFT, Polyphen-2, and MutationTaster** |
| --- | --- | --- | --- | --- | --- |
| chr1 | 110738300 | *SLC6A17* | A>C | None | 0.03, 0.999, 0.999 (D) |
| chr1 | 155448357 | *ASH1L* | T>C | None | 0.5, 0.851, 1 (D) |
| chr1 | 161161952 | *ADAMTS4* | G>A | None | 0.1, 0.999, 1 (D) |
| chr2 | 69177269 | *GKN2* | C>A | None | 0, 1, 0.998 (D) |
| chr2 | 227875190 | *COL4A4* | C>A | Alport syndrome, autosomal recessive; Hematuria, familial benign | 0, 1, 1 (D) |
| chr3 | 44926943 | *TGM4* | A>C | None | 0.03, 0.943, 0 (P) |
| chr3 | 119305201 | *ADPRH* | A>C | None | 0.03, 0.844, 0.996 (D) |
| chr5 | 102440292 | *GIN1* | G>T | None | 0.04, 0.994, 0.993 (D) |
| chr5 | 140166025 | *PCDHA1* | G>C | None | 0, 1, 0.615 (D) |
| chr6 | 10989970 | *ELOVL2* | G>A | None | 0.16, 1, 1 (D) |
| chr6 | 167728725 | *UNC93A* | T>C | None | 0.02, 1, 1 (D) |
| chr7 | 120704315 | *CPED1* | G>C | None | 0.04, 0.933, 1 (D) |
| chr8 | 9437701 | *TNKS* | C>G | None | 0.08, 0.992, 1 (D) |
| chr9 | 27284950 | *EQTN* | T>A | None | 0, 0.893, 0 (P) |
| chr9 | 79469075 | *PRUNE2* | G>A | None | 0.01, 1, 1 (D) |
| chr9 | 104162194 | *ZNF189* | T>G | None | 0.04, 0, 1 (D) |
| chr9 | 139910045 | *ABCA2* | C>T | None | 0, 1, 0.998 (D) |
| chr10 | 12126655 | *DHTKD1* | A>G | 2-aminoadipic 2-oxoadipic aciduria; Charcot-Marie-Tooth disease, axonal, type 2Q | 0, 0.474, 0.144 (P) |
| chr10 | 34558751 | *PARD3* | G>A | None | 0.01, 1, 1 (D) |
| chr10 | 104140341 | *GBF1* | C>T | None | 0.02, 0.975, 1 (D) |
| chr11 | 117163847 | *BACE1* | G>A | None | 0.01, 1, 1 (D) |
| chr12 | 53587136 | *ITGB7* | C>T | None | 0.02, 1, 1 (D) |
| chr15 | 55965826 | *PRTG* | T>C | None | 0.06, 1, 1 (D) |
| chr15 | 75015215 | *CYP1A1* | T>G | None | 0, 0.989, 1 (D) |
| chr16 | 57416775 | *CX3CL1* | C>T | None | 0, 1, 0.985 (D) |
| chr17 | 7916570 | *GUCY2D* | G>A | Cone-rod dystrophy 6; Leber congenital amaurosis 1 | 0.02, 1, 1 (D) |
| chr17 | 33504518 | *UNC45B* | A>T | None | 0.36, 1, 1 (D) |
| chr18 | 7024403 | *LAMA1* | C>G | None | 0, 1, 1 (D) |
| chr19 | 1005209 | *GRIN3B* | T>A | None | 0, 0.916, 0.982 (D) |
| chr19 | 44652952 | *ZNF234* | T>G | None | 0, 0.997, 1 (D) |
| chrX | 129203602 | *ELF4* | A>C | None | 0, 0.875, 1 (D) |
| chrX | 136652155 | *ZIC3* | A>C | Congenital heart defects, nonsyndromic, 1, X-linked | 0.32, 0.971, 1 (D) |
| chr3 | 121514370 | *IQCB1* | insG | Senior-Loken syndrome 5 | NA, NA, 1 (D) |
| chr6 | 99936648 | *USP45* | delTC | None | NA, NA, 1 (D) |
| chr11 | 18478272 | *LDHAL6A* | delG | None | NA, NA, 1 (D) |
| chr12 | 51740416 | *CELA1* | insG | None | NA, NA, 1 (D) |
| chr13 | 48615239 | *NUDT15* | insG | None | NA, NA, 1 (D) |

Sift score≤0.05 indicates deleterious; Polyphen-2 score≥0.453 indicates damaging; NA, not available; D, disease-causing predicted by Mutationtaster; P, polymorphism predicted by Mutationtaster.

**Table S6.**

**Functional enrichment for patients (Ⅰ-2 and Ⅱ-2) in family 1. No functional categories are enriched after multiple comparison correction.**

| **Patient** | **Term** | **Count** | **Genes** | **Fold**  **Enrichment** | **P Value** | **Bonferroni** |
| --- | --- | --- | --- | --- | --- | --- |
| Ⅰ-2 | signal | 8 | *NELL1,* *NOMO1,* *F5,* *FBLN1,* *GABBR2,* *MSLN,* *NRXN2,* *ERBB2* | 1.4 | 3.2E-1 | 1.0E0 |
|  | transmembrane | 11 | *ABCA10,* *NOMO1,* *GALNT15,* *GABBR2,* *NRXN2,* *OR5H14,* *OR5H2,* *PHEX,* *KCNQ3,* *KCNQ4,* *ERBB2* | 1.3 | 3.4E-1 | 1.0E0 |
|  | cell junction | 3 | *INADL,* *GABBR2,* *PKP2* | 4.3 | 1.5E-1 | 1.0E0 |
|  | egf-like domain | 3 | *NELL1,* *FBLN1,* *NRXN2* | 7.4 | 5.9E-2 | 1.0E0 |
| Ⅱ-2 | signal | 15 | *ADAMTS4,* *BACE1,* *CX3CL1,* *CPED1,* *EQTN,* *CELA1,* *COL4A4,* *GKN2,* *GRIN3B,* *GUCY2D,* *ITGB7,* *LAMA1,* *PARD3,* *PCDHA1,* *PRTG* | 2.4 | 1.4E-3 | 1.6E-1 |
|  | transmembrane | 13 | *ABCA2,* *BACE1,* *CX3CL1,* *EQTN,* *ELOVL2,* *GRIN3B,* *GUCY2D,* *ITGB7,* *PARD3,* *PCDHA1,* *PRTG,* *SLC6A17,* *UNC93A* | 1.4 | 2.0E-1 | 1.0E0 |
|  | cell adhesion | 4 | *CX3CL1,* *ITGB7,* *LAMA1,* *PCDHA1* | 4.9 | 4.4E-2 | 1.0E0 |
|  | transcription regulation | 5 | *ELF4,* *ZIC3,* *ASH1L,* *ZNF189,* *ZNF234* | 1.3 | 5.3E-1 | 1.0E0 |

**Table S7. Analytic results of candidate variants obtained from exome sequencing for family 2**

| **Chromosome** | **Position** | **Substitution** | **Gene Name** | **Gene-Phenotype by OMIM** | **Score from SIFT, Polyphen-2, and MutationTaster** |
| --- | --- | --- | --- | --- | --- |
| **Homozygous candidate variants:** | | | | | |
| chr2 | 131221170 | C>T | *POTEI* | None | 0, NA, 1 (P) |
| chr17 | 39471763 | C>T | *KRTAP17-1* | None | 0, 0.994, 0 (P) |
| chrX | 2856155 | C>T | *ARSE* | Chondrodysplasia punctata, X-linked recessive | 0.43, 0.939, 0.001 (P) |
| **Compound heterozygous candidate variants:** | | | | | |
| chr11 | 2185599 | G>A | ***TH*** | Segawa syndrome, recessive | 0, 1, 1 (D) |
| chr11 | 2187929 | C>T | ***TH*** | Segawa syndrome, recessive | 0, 1, 1(D) |
| chr2 | 152348947 | G>T | *NEB* | Nemaline myopathy 2, autosomal recessive | 0.13, 1, 1 (D) |
| chr2 | 152548631 | C>T | *NEB* | Nemaline myopathy 2, autosomal recessive | 0.19, 1, 1 (D) |
| chr2 | 179414440 | A>G | *TTN* | Cardiomyopathy, dilated, 1G; Cardiomyopathy, familial hypertrophic, 9 | 0, 0.801, 0.935 (D) |
| chr2 | 179500421 | A>C | *TTN* | Cardiomyopathy, dilated, 1G; Cardiomyopathy, familial hypertrophic, 9 | 0, 0.919, 1 (D) |
| chr2 | 179614801 | G>A | *TTN* | Cardiomyopathy, dilated, 1G; Cardiomyopathy, familial hypertrophic, 9 | 0.59, 0.983, 1 (D) |
| chr5 | 156766081 | T>G | *CYFIP2* | None | 0, 0.977, 1 (D) |
| chr5 | 156766084 | A>G | *CYFIP2* | None | 0.02, 0.775, 1 (D) |
| chr7 | 151927025 | A>G | *KMT2C* | None | 0, 1, 1 (D) |
| chr7 | 151945007 | C>T | *KMT2C* | None | 0.43, 1, 1 (D) |
| chr7 | 151970951 | C>T | *KMT2C* | None | 0.04, 0.984, 1 (D) |
| chr17 | 45234303 | G>C | *CDC27* | None | 0.16, 0.799, 1 (D) |
| chr17 | 45234417 | A>G | *CDC27* | None | 0.56, 0.131, 1 (D) |
| chr17 | 45234707 | T>A | *CDC27* | None | 0.07, 0.624, 1 (D) |
| chrX | 135956462 | G>C | *RBMX* | None | 0.01, 0.889, 0 (P) |
| chrX | 135958704 | G>C | *RBMX* | None | 0.03, 0.627, 0 (P) |
| **Heterozygous candidate variants:** | | | | | |
| **Chromosome** | **Position** | **Substitution** | **Gene Name** | **Gene-Phenotype by OMIM** | **Score from SIFT, Polyphen-2, and MutationTaster** |
| chr1 | 10705025 | C>G | *CASZ1* | None | 0.1, 1, 1 (D) |
| chr1 | 45810894 | T>C | *TESK2* | None | 0.04, 0.919, 1 (D) |
| chr1 | 62579810 | T>G | *INADL* | None | 0, 0.998, 1 (D) |
| chr1 | 143767547 | G>A | *PPIAL4G* | None | 0.03, 0.999, 0.079 (P) |
| chr1 | 145601821 | G>C | *POLR3C* | None | 0, 0.055, 0.338 (P) |
| chr1 | 150808896 | A>C | *ARNT* | Leukemia, acute myeloblastic | 0, 1, 1 (D) |
| chr1 | 153911500 | C>G | *DENND4B* | None | 0.03, 1, 1 (D) |
| chr1 | 176708766 | G>T | *PAPPA2* | None | 0.52, 0.057, 1 (D) |
| chr1 | 205689724 | G>C | *NUCKS1* | None | 0.04, 0.99, 1 (D) |
| chr1 | 234743106 | T>C | *IRF2BP2* | None | 0, 1, 1 (D) |
| chr1 | 238049121 | G>T | *ZP4* | None | 0.16, 0.998, 0 (P) |
| chr2 | 74784003 | A>C | *DOK1* | None | 0.02, 0.97, 1 (D) |
| chr2 | 168101695 | A>G | *XIRP2* | None | 0.09, 0.995, 0.998 (D) |
| chr2 | 203395663 | G>C | *BMPR2* | Pulmonary hypertension, familial primary, 1, with or without HHT; Pulmonary hypertension, primary, fenfluramine or dexfenfluramine-associated | 0.25, 0.994, 1 (D) |
| chr2 | 219563485 | A>C | *STK36* | None | 0.05, 0.997, 0.865 (D) |
| chr2 | 228172515 | T>C | *COL4A3* | Alport syndrome, autosomal dominant; Alport syndrome, autosomal recessive | 0.77, 0.999, 1 (D) |
| chr3 | 9515182 | C>T | *SETD5* | Mental retardation, autosomal dominant 23 | 0.03, 0.4, 0.362 (P) |
| chr3 | 39452384 | A>C | *RPSA* | Asplenia, isolated congenital | 0, 0.001, 1 (D) |
| chr3 | 47960310 | G>C | *MAP4* | None | 0, 0.935, 0.005 (P) |
| chr3 | 122433259 | T>C | *PARP14* | None | 0.01, 1, 1 (D) |
| chr4 | 2242253 | C>T | *HAUS3* | None | 1, 0.892, 0 (P) |
| chr4 | 39303988 | A>C | *RFC1* | None | 0.01, 1, 1 (D) |
| chr4 | 70074101 | T>C | *UGT2B11* | None | 0.03, 0.877, 0 (P) |
| chr4 | 190874234 | C>T | *FRG1* | None | 0.02, 0.998, 1 (D) |
| chr5 | 16481165 | G>A | *FAM134B* | Neuropathy, hereditary sensory and autonomic, type IIB | 0.01, 1, 0.992 (D) |
| chr5 | 64483914 | G>A | *ADAMTS6* | None | 0, 1, 1 (D) |
| chr5 | 93800608 | C>G | *KIAA0825* | None | 0.01, 0.997, 0.998 (D) |
| chr5 | 139745583 | G>T | *SLC4A9* | None | 0, 0.999, 1 (D) |
| chr6 | 25610319 | C>G | *LRRC16A* | None | 0.34, 0.919, 1 (D) |
| chr6 | 25813428 | A>C | *SLC17A1* | None | 0, 0.999, 1 (D) |
| chr6 | 38565732 | A>C | *BTBD9* | None | 0.11, 0.991, 1 (D) |
| chr6 | 42974804 | C>T | *PPP2R5D* | None | 0, 0.999, 1 (D) |
| chr6 | 44148398 | A>G | *CAPN11* | None | 0.04, 0.822, 0.733 (D) |
| chr6 | 121563477 | C>A | *TBC1D32* | None | 0.07, 0.998, 1 (D) |
| chr6 | 133782273 | G>A | *EYA4* | Cardiomyopathy, dilated, 1J; Deafness, autosomal dominant 10 | 0.18, 0.999, 1 (D) |
| chr7 | 98478802 | C>T | *TRRAP* | None | 0.04, 0.997, 1 (D) |
| chr7 | 131128355 | C>T | *MKLN1* | None | 0.25, 0.728, 1 (D) |
| chr7 | 135263559 | G>A | *NUP205* | None | 0.16, 0.87, 1 (D) |
| chr7 | 142723621 | A>C | *OR9A2* | None | 0.04, 0.629, 0 (P) |
| chr8 | 56708574 | A>G | *TGS1* | None | 0.04, 0.704, 0.019 (P) |
| chr8 | 94746730 | G>A | *RBM12B* | None | 0, 1, 0 (P) |
| chr9 | 107645430 | C>G | *ABCA1* | HDL deficiency, type 2; Tangier disease | 0, 1, 1 (D) |
| chr9 | 111903823 | A>C | *FRRS1L* | None | 0.07, 1, 1 (D) |
| chr9 | 111979351 | G>A | *EPB41L4B* | None | 0.01, 0.957, 1 (D) |
| chr10 | 73545427 | C>T | *CDH23* | Deafness, autosomal recessive 12; Usher syndrome, type 1D | 0.1, 0.998, 1 (D) |
| chr10 | 105108744 | G>C | *PCGF6* | None | 0.09, 0.999, 0.999 (D) |
| chr11 | 126201321 | A>C | *DCPS* | None | 0.09, 1, 1 (D) |
| chr12 | 22676453 | A>C | *C2CD5* | None | 0, 1, 1 (D) |
| chr12 | 39735912 | T>C | *KIF21A* | Fibrosis of extraocular muscles, congenital, 1;  Fibrosis of extraocular muscles, congenital, 3B | 0.04, 0.115, 0.936 (D) |
| chr12 | 46598200 | C>T | *SLC38A1* | None | 0.36, 0.65, 1 (D) |
| chr12 | 55968483 | C>G | *OR2AP1* | None | 0, NA, 1 (D) |
| chr12 | 70150426 | C>G | *RAB3IP* | None | 0.04, 0.998, 1 (D) |
| chr13 | 24798487 | C>T | *SPATA13* | None | 0, NA, 1 (D) |
| chr13 | 50243942 | G>A | *EBPL* | None | 0.23, 0.925, 1 (D) |
| chr14 | 73683938 | G>T | *PSEN1* | Alzheimer disease, type 3 | 0.06, 0.97, 1 (D) |
| chr15 | 65862519 | T>C | *PTPLAD1* | None | 0, 1, 1 (D) |
| chr15 | 78786487 | G>T | *IREB2* | None | 0.73, 1, 1 (D) |
| chr16 | 15703403 | C>T | *KIAA0430* | None | 0.09, 0.999, 1 (D) |
| chr16 | 21038368 | A>G | *DNAH3* | None | 0, 1, 1 (D) |
| chr16 | 24582685 | G>A | *RBBP6* | None | 0.05, 0.001, 0.612 (D) |
| chr16 | 66967614 | A>C | *FAM96B* | None | 0.01, 0.999, 1 (D) |
| chr16 | 70926362 | C>G | *HYDIN* | Ciliary dyskinesia, primary, 5 | 0, 1, 1 (D) |
| chr16 | 72143360 | A>G | *DHX38* | None | 0, 0.999, 1 (D) |
| chr16 | 88786226 | G>C | *PIEZO1* | Dehydrated hereditary stomatocytosis with or without pseudohyperkalemia and/or perinatal edema | 0.4, 0.508, 1 (D) |
| chr17 | 5036214 | C>T | *USP6* | None | 0, 0.899, 0.001 (P) |
| chr17 | 6021383 | A>G | *WSCD1* | None | 0.19, 0.998, 1 (D) |
| chr17 | 18232153 | C>A | *SHMT1* | None | 0, 0.941, 0.951 (D) |
| chr17 | 38564871 | A>C | *TOP2A* | DNA topoisomerase II, resistance to inhibition of, by amsacrine | 0, 0.273, 1 (D) |
| chr17 | 41165343 | C>T | *IFI35* | None | 0.01, 0.999, 0 (P) |
| chr17 | 78353498 | G>A | *RNF213* | Moyamoya disease 2, susceptibility to | 0.04, 1, 0.778 (D) |
| chr18 | 54362424 | G>C | *WDR7* | None | 0.39, 0.993, 1 (D) |
| chr19 | 48806993 | A>C | *CCDC114* | Ciliary dyskinesia, primary, 20 | 0.04, 0.999, 0.993 (D) |
| chr19 | 49671196 | C>T | *TRPM4* | Progressive familial heart block, type IB | 0, 1, 1 (D) |
| chr22 | 24579157 | G>T | *SUSD2* | None | 0.31, 1, 1 (D) |
| chr22 | 36712653 | A>C | *MYH9* | Deafness, autosomal dominant 17; Epstein syndrome | 0, 0.992, 1 (D) |
| chr22 | 39497312 | A>C | *APOBEC3H* | None | 0, 0.997, 0 (P) |
| chrX | 30872292 | C>T | *TAB3* | None | 0.73, 1, 1 (D) |
| chr1 | 179064271 | delTGTA | *TOR3A* | None | NA, NA, 1 (D) |
| chr2 | 152107578 | delTCTC | *RBM43* | None | NA, NA, 1 (D) |
| chr18 | 14513709 | delAGTC | *POTEC* | None | NA, NA, 1 (D) |
| chr21 | 45959558 | delTC | *KRTAP10-1* | None | NA, NA, 1 (D) |

Sift score≤0.05 indicates deleterious; Polyphen-2 score≥0.453 indicates damaging; NA, not available; D, disease-causing predicted by Mutationtaster; P, polymorphism predicted by Mutationtaster.
